# Supplementary material for: Glycyrrhizic Acid Inhibits High-Mobility Group Box-1 and Homocysteine-Induced Vascular Dysfunction
Source: Nutrients. 2023 Jul 18;15(14):3186. doi: 10.3390/nu15143186 (PMC10383373; doi:10.3390/nu15143186)
Supplement: Supplementary file 1 [file nutrients-15-03186-s001.zip › nutrients-2457687-supplementary.pdf]

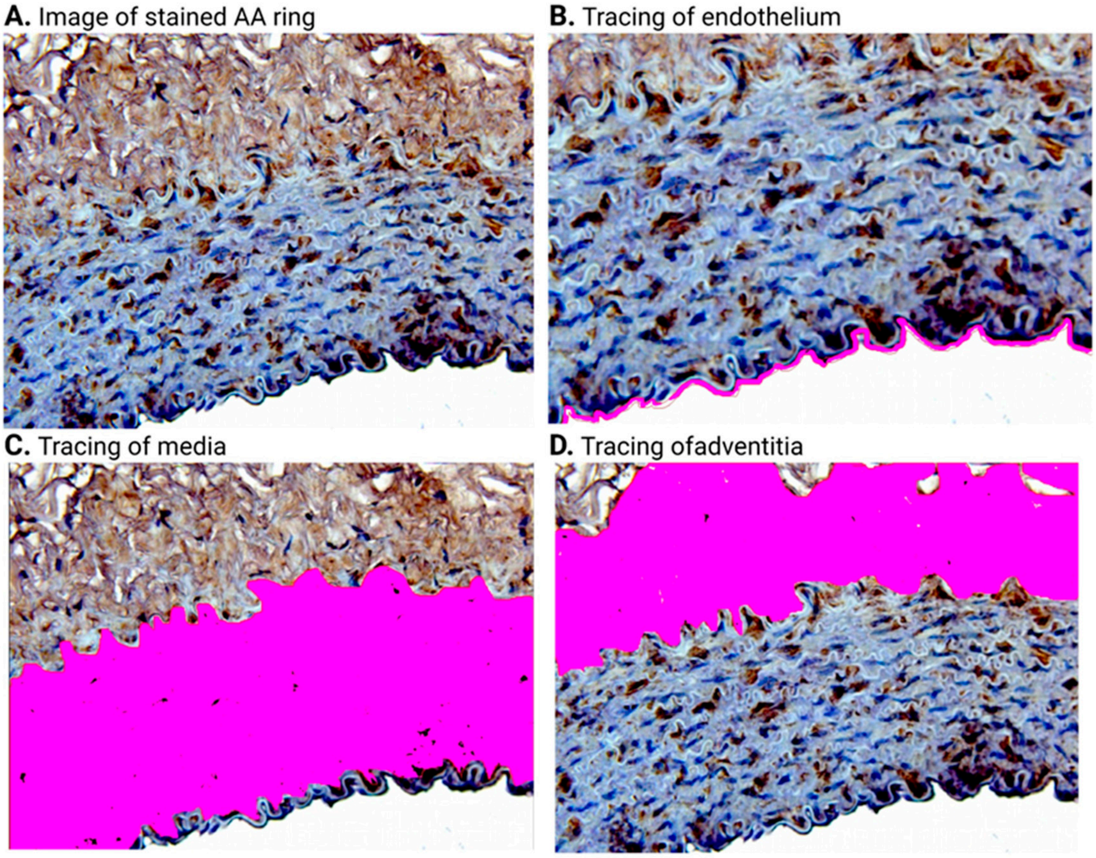

Supplementary Figure S1: Identification of different vascular layers. A. Image of AA coronal section from a rabbit fed our 4-week AD. **B.** Image that has been traced using the MCID program ribbon tool and is highlighting the endothelium (depicted by magenta color). **C.** Image that has been traced using the MCID program outline tool and is highlighting the media layer (depicted in magenta). **D.** Image that has been traced using the MCID program outline tool and is highlighting the adventitia layer (depicted in magenta). *Abbreviations: AA*, abdominal aorta; *AD*, atherogenic diet.
